# Perspectives on ageing, later life and ethnicity: ageing research in ethnic minority contexts

**DOI:** 10.1017/S0144686X14001536

**Published:** 2015-05

**Authors:** MARIA ZUBAIR, MERIEL NORRIS

**Affiliations:** * School of Sociology and Social Policy, University of Nottingham, UK.; † Centre for Dementia, Institute of Mental Health, University of Nottingham, UK.; ‡ College of Health and Life Sciences, Brunel University London, UK.; § Institute of Healthy Ageing, Brunel University London, UK.

**Keywords:** ageing, ethnicity, gerontology, *othering*, research agendas, social inequalities

## Abstract

This special issue focuses broadly upon questions and themes relating to the current conceptualisations, representations and use of ‘ethnicity’ (and ethnic minority experiences) within the field of social gerontology. An important aim of this special issue is to explore and address the issue of ‘otherness’ within the predominant existing frameworks for researching those who are ageing or considered aged, compounded by the particular constructions of their ethnicity and ethnic ‘difference’. The range of theoretical, methodological and empirical papers included in this collection provide some critical insights into particular facets of the current research agendas, cultural understandings and empirical focus of ethnic minority ageing research. The main emphasis is on highlighting the ways in which ethnic cultural homogeneity and ‘otherness’ is often assumed in research involving older people from ethnic minority backgrounds, and how wider societal inequalities are concomitantly (re)produced, within (and through) research itself – for example, based on narrowly defined research agendas and questions; the assumed age and/or ethnic differences of researchers *vis-à-vis* their older research participants; the workings of the formalised ethical procedures and frameworks; and the conceptual and theoretical frameworks employed in the formulation of research questions and interpretation of data. We examine and challenge here the simplistic categorisations and distinctions often made in gerontological research based around research participants' ethnicity, age and ageing and assumed cultural differences. The papers presented in this collection reveal instead the actual complexity and fluidity of these concepts as well as the cultural dynamism and diversity of experiences within ethnic groups. Through an exploration of these issues, we address some of the gaps in existing knowledge and understandings as well as contribute to the newly emerging discussions surrounding the use of particular notions of ethnicity and ethnic minority ageing as these are being employed within the field of ageing studies.

## Introduction

The demographic landscape of Europe, and the Western world more generally, is changing significantly as a consequence of population ageing and immigration (Nguyen 2011; Warnes *et al.* 2004). With the overall ageing of the general population and the increasing numbers of immigrants reaching old age in their host countries, racial and ethnic minorities are now beginning to constitute the fastest growing segment of the elderly population within many Western countries (Burholt 2004; Jimenez *et al.* 2012). This recent as well as further expected future growth in the number of older people from ethnic minority backgrounds calls for the need to recognise, uncover and understand the diverse and heterogeneous nature of old age and later life experiences and needs within these Western host countries (*see* Manthorpe 2010; Victor, Martin and Zubair 2012; Vincent, Phillipson and Downs 2006). However, academic research which seeks to examine the experiences of ageing among ethnic minority older people, or even which pays attention to issues relating to ethnicity in old age more generally, remains scarce (Blakemore 1999; Blakemore and Boneham 1994; Phillipson 2015; Wray 2003*a*; *see also* Warnes *et al.* 2004).

This special issue of *Ageing & Society* is an attempt to reinforce the importance of a greater level of engagement with issues relating to ethnicity within ageing studies and social gerontology. Such an engagement requires a sustained concern with, and increased efforts towards, devoting more research capacity to the study of ethnicity and ethnic minority older people in social gerontology. Moreover, this engagement needs to be understood not in the narrow sense of merely a greater inclusion and visibility of ethnic minority older people within ageing research. While such inclusion is undoubtedly important, there is also a need for focusing a more critical eye on the currently dominant research agendas, frameworks and trends with respect to ethnic minority ageing research, in terms of how ethnic minority ageing and older people are conceptualised, understood and represented within this smaller body of gerontological literature (*see* Phillipson 2015; Torres 2015; Zubair and Victor 2015). This critical appraisal of the current research frameworks should involve identification of new ways of looking at and understanding ethnicity in older age. More specifically, this should involve a recognition of the need to move away from the more rigid categorisations and essentialist understandings of ‘ethnicity’ and ethnic ‘difference’, as well as age and ageing, and an appreciation instead of the diversity of experiences, issues and needs among ethnic minority older people as with older people more generally (*see* Iliffe and Manthorpe 2004; Torres 2015).

The papers in this special issue result from the Economic and Social Research Council (ESRC) seminar series on ‘Ageing, Race and Ethnicity’, held in the United Kingdom (UK) during the period 2012–2014. The seminar series sought to explore, develop and theorise our knowledge and understandings of the experiences of ageing and later life amongst ethnic minority communities. Hosted by Brunel University and the Brunel Institute for Ageing Studies (BIAS) – with a wide range of seminars organised at Brunel University, Swansea University and the University of Worcester in the UK, this seminar series brought together a diverse group of speakers and attendees to share their perspectives, experiences and voices in relation to ethnic minority ageing research. The participants included academics from a range of disciplines (including UK-based and international experts in the fields of ethnicity and social gerontology and those at various stages of their research careers), policy makers, health and social care practitioners, commissioners of services, and those working in the voluntary and statutory sectors. This diverse group of participants together contributed to some lively discussions by sharing their own varied perspectives, the theoretical and methodological approaches they have been developing, their empirical findings, and the policy and practice implications of the current research in the field. An important outcome of this seminar series was the articulation of a clear need to examine both the conceptualisations of ‘ethnicity’ within social gerontology and the current practices of researching ethnic minority older people's experiences.

Research on ethnic minority older people's ageing and later-life experiences continues to remain, without a doubt, limited in breadth as well as under-theorised – with specific notions of ‘ethnicity’ and ‘age/ageing’ continuing to define the dominant thinking within the field. However, some of these emerging discussions and critical insights which were presented at the seminar series, and hence are beginning to come to the fore, will hopefully open up new horizons and directions for the further development of our knowledge and understandings in the area of ethnicity in later life and older age. Following the success of the seminar series, this special issue is a subsequent attempt to capture in writing, reflect upon, and further explore and interrogate our current and emerging understandings and theorisations of ‘ethnicity’ and ethnic minorities' later-life experiences in the field of ageing studies.

## Researching ageing among ethnic minorities – significance, trends and future directions

The particular ageing and later-life experiences of members of ethnic minority communities in the Western world can be identified as deserving special attention because of these minorities' often distinctive situations in terms of the higher levels of disadvantages, inequalities and exclusions experienced into old age compared with the dominant White ethnic majorities (*see* Nazroo 2006; Nazroo *et al.* 2004; Phillipson *et al.* 2000; Yu 2000). Within their status as ethnic minorities and migrants, many of these populations experience living in countries where the dominant social policies, norms and discourses, in addition to the particular material and economic inequalities experienced, often further marginalise them by defining and constructing their specific experiences (including those of old age) in terms of their cultural difference or ethnic ‘otherness’ (Torres 2006; Warnes *et al.* 2004; *see also* Zubair and Victor 2014, 2015). As Torres (2006) notes in the case of ethnic minority older people in Sweden, the type of attention received by this group, for example, has involved recognising or labelling them in terms of a specific homogenised social category, that of ‘elderly immigrants’. Such categorisation by those concerned with elderly care and policy, she argues, has led to these ethnic minority older people being constructed and perceived of as a social problem. Hence, even as the recent concern with regards to the demographic ageing of ethnic minority populations is encouraging governments, social policy circles and academics to further shift their gaze towards issues of ageing and later life among ethnic minorities, the focus of this attention at large can be seen as being very limited so far – concentrating mainly on those aspects of the ageing experience and sections of older ethnic minority populations which have been perceived as being problematic and requiring social policy or practice interventions (Torres 2006; Victor, Zubair and Martin in press). Furthermore, often the explanations for the problems experienced by these groups are described as being located in their specific ethnic cultural norms and differences and thus their ‘ethnicity’ as opposed to the actual social context of cumulative disadvantages and social inequalities experienced by these groups over the lifecourse (Nazroo 2004, 2011; *see also* Brotman 2003). Hence, ethnic categories continue to be reified with an accompanying neglect of other facets of ethnic minority older people's identity and difference/similarity (*see* Iliffe and Manthorpe 2004; Nazroo 2011), and ethnic minority older people are often still perceived of, treated and represented as the culturally static and homogeneous ‘others’ (Torres 2006). The actual cultural diversity and complexity of experiences among older people (and those in later life) belonging to any ethnic minority group, resulting from cross-cutting, overlapping and changing social locations and identifications (*see* Torres 2001; Zubair, Martin and Victor 2012*a*), on the other hand, remains less well-documented within gerontological research and literature.

At the same time as the older trends continue, there is now also an increased recognition of the importance for academic thinking and research in the field of social gerontology to move beyond the ethnocentric White cultural lens (*see* Torres 1999; Wray 2003a, 2003b) and the more problem-focused and essentialist understandings and conceptualisations that it tends to advance in relation to ethnicity (*see* Torres 2006, 2015). Hence, rather than treating ethnicity as well as age and ageing as being fixed, static and discrete categories, these need to be conceptualised and understood instead as being socially constructed and therefore as being fluid, changing and context-dependent (*see* Norris 2015; Torres 2015; Zubair, Martin and Victor 2012*a*).

With respect to age/ageing identities, social gerontology has for quite some time acknowledged the socially constructed nature of these categories and shown how old age and later life are socially (and structurally) constituted, represented and understood, often to the detriment of older people (*see* Estes 1986; Phillipson 1982; Townsend 1981; Walker 1981). There are also many examples of sustained efforts involving social gerontologists, sociologists, anthropologists and socio-linguists at dismantling the essentialism which continues to be characteristic of some of the current writing and thinking on ageing and older people (*see* Coupland 2000; Kaufman 1994; Pickard 2013, 2014*a*, 2014*b*; Woodward 2003), with scholars advancing critical perspectives focusing upon how older people as a social group continue to occupy disadvantaged positions in society and have also often come to be positioned and understood as the problematic ‘others’ (*see* Biggs 2001; Leontowitsch 2012; Russell 1999). Speaking from a socio-linguistic perspective on ageing, and emphasising the heterogeneity of older people and later-life experiences to the *Ageing & Society* readership, Coupland aptly notes the fluidity and instability of age-related social categories (*see also* Nikander 2009):

…the social categories to which we are accustomed (as both researchers and lay observers and participants in social life) do not have the coherence and stability that we tend to assume. The ways in which we invoke ‘being 50’, for example, are often complex and may be mitigated or carefully contextualised. Many constructions are provisional expressions during extended negotiated sequences, and others are for some particular immediate purpose; whichever, they are unlikely to be full or final accounts of the meaning of being 50 … A commitment to avoid over-determining age is probably an appropriate principle and prerequisite for gerontological research: as understanding of ageing and old age grows, an awareness of heterogeneity slowly supplants broad characterisations. While readers of this journal will be well informed about the real and sometimes depressingly real social correlates of old age, they will also be aware of the risk of over-generalising about age and the links between life-stage and experience in an increasingly complex and fluid social world. (Coupland 2009: 852–3)

Notwithstanding the wider recognition within social gerontology about the socially constructed nature of age/ageing identities, the instability and fluidity of age/ageing categories, and the diversity of older people and later-life experiences, this understanding appears to remain far less developed and not fully articulated within the majority of the ageing research and scholarly literature which focuses upon ethnic minority older people. For this group of older people, the continued predominance of essentialist notions of ‘ethnicity’ within the gerontological literature has meant that their ageing and later-life experiences also continue to be conceptualised and presented largely in homogenised and *othering* terms (Torres 2015). The ethnicity literature and scholarship itself has, on the other hand, long challenged essentialist notions of ethnicity, race and culture (*see* Barth 1969; Cornell and Hartmann 1998; Craig *et al.* 2012; Elrick, Schneiderhan and Khan 2014; Hall 1992; Nagel 1994; Song 2003; Waters 1990; Wimmer 2008*a*, 2008*b*). Contrary to the dominant trend within social gerontology with respect to the study of ethnicity, within the latter literature, ethnicity is understood as a ‘dynamic, constantly evolving property of both individual identity and group organisation’ and as a product of actions undertaken by groups as they shape and reshape their own self-definition and culture in interaction with others, as well as shaping and reshaping ethnic categories and definitions for others (Nagel 1994: 152). Ethnicity, identity and ‘ethnic culture’ are, hence, perceived within the mainstream (or rather, non-gerontological) ethnicity literature to be socially constructed, relational, situational, fluid and dynamic – shifting, changing and transforming over time, space and context – and as emergent processes involving ongoing negotiations and redefinitions, rather than being fixed and static entities that individuals are deemed to possess (Craig *et al.* 2012; Elrick, Schneiderhan and Khan 2014; Song 2003; Waters 1990; Wimmer 2008*a*, 2008*b*). More recently, inspired by feminist theory and gender studies, ethnicity scholarship has moved further to explore and develop also the concept of intersectionality (*see* Bürkner 2012; Crenshaw 1991; Denis 2008). This has involved an acknowledgement of the interconnections and interplays between an individual's multiple social identifications, and the mapping out of the implications of multiple social differentiations for the individual (including those linked with the potentially contradictory social locations and positionings of some individuals across the varied social hierarchies and axis of power/subordination, privilege/stigma and inclusion/exclusion) (*see* Anthias 1998; Ratna 2013; Yuval-Davis 2006).

Linked with the above-mentioned developments in ethnicity scholarship, particularly the recognition of the instability of ‘ethnicity’ as a category, ethnicity scholars have also further begun to interrogate the extent to which ‘ethnicity’ and ethnic categories are in themselves useful as conceptual tools or frameworks for analysis, explanation and theorisation (*see* Bradby 1995, 2003; Carter and Fenton 2009; Elrick, Schneiderhan and Khan 2014; Nazroo 2011; Vidas and Hoffmann 2012). Such emerging discussions regarding the utility of ‘ethnicity’ and how it is conceptualised and used within social policy, practice and research (as well as in public discourses more generally), together with the presently dominant perspectives of social constructivism and intersectionality within ethnicity scholarship have much to offer, as Torres (2015) suggests, to social gerontology. Inclusion of such perspectives within social gerontology is likely to broaden and deepen our current understandings in relation to the later-life situations and experiences of ethnic minority older people. More crucially, an embracement of these perspectives (along with the non-essentialist understandings that these promote in relation to both ethnicity and ethnic minority later life and ageing) would be an important step towards addressing and countering the *othering* assumptions and practices that currently continue to prevail with respect to ethnic minority older people within the field of social gerontology.

It can be argued that the *othering* assumptions and practices within the gerontological literature and research involving ethnic minority older people are currently present and operate in a number of ways to (re)create and reproduce disadvantages and social inequalities for this group of older people within (and through) research itself (Zubair and Victor 2015). Moreover, not only are these *othering* assumptions and practices apparent in the current tendencies towards not sufficiently acknowledging and addressing within research the internal social and cultural diversity and heterogeneity of experiences existing within any ethnic minority group (or even potential overlaps in situations and experiences with the general populations), but also in the continued predominance of a more problem-focused approach in studying and understanding ethnic minority older people's ageing and later-life experiences. The ways in which research agendas and questions, and even research methodologies and processes, are currently framed for research with this group of older people, for example, involves a particular focus (even if implicitly) on the perceived problematic aspects of their ageing and vulnerability – such as greater ill-health and lack of consultation, engagement and access to services. This positions these older people and their ethnicity in terms of their deviance from the desired, standard, social and cultural norms that are perceived to be typical of the general population, hence constructing these older people and their ethnicity as problematic (*see* Brotman 2003; Zubair and Victor 2015). In this respect, while a neglect of the wider, non-problematic, aspects of ageing and old age among ethnic minority groups has erroneously constructed these ethnic minority older people as a particularly vulnerable and deviant social grouping, an over-emphasis on their ‘ethnicity’, encapsulated in the discourse of ‘special needs’ and cultural ‘differences’, has further worked to marginalise this older group of people by constructing their ethnicity in negative terms, perceived as being responsible for their disadvantaged positions into old age (Brotman 2003; Sin 2004; Torres 2006). This is at the same time as other important aspects of their personal and social identities, and the wider context of the systematic social inequalities and disadvantages experienced by these older people within their everyday lives and social worlds has often been overlooked.

Pointing to the limited focus within social gerontology on the wider, non-problematic, aspects of ethnicity and ethnic minority ageing, Victor, Zubair and Martin (in press) explain the lack of crossover between the literatures from the previously two distinct fields of study – *i.e.* that of racial, ethnic and migration studies and classical social gerontology. They describe how the focus of social gerontology in relation to ethnic minority ageing remained concentrated until very recently on specific problem areas such as access to health-care facilities and needs assessments of ethnic minority older people, quality of life in old age, the relationships between informal and formal care services, the support needs and experiences of informal carers, and so on (*see* Giuntoli and Cattan 2012; Lawrence *et al.* 2008). Consideration of the wider aspects of the ageing experience among ethnic minorities, particularly over the lifecourse, on the other hand, has been paid very scant attention until very recently (*see also* Phillipson 2015). Concomitantly, the body of literature falling within the mainstream of the field of race and ethnicity or focusing on migrant, ethnic minority, populations has until now, with a few rare exceptions (*see* Gardner 2002; Qureshi 1998), failed to include issues of ageing and later life or to address the impact of potentially disadvantaged (or at least distinctive) racial and ethnic or minority locations on the ageing or later-life issues and experiences of ethnic minorities. The preoccupation within the mainstream literature on race, ethnicity and migration has so far been mainly with issues relating to the social integration of ethnic minorities. Within this, while one key area of academic research and thinking has focused upon issues relating to the disadvantages, inequalities and exclusions faced by ethnic minority groups within the areas of education, employment, housing, health, and so on (*see* Mason 2000; Modood *et al.* 1997), the other has been preoccupied with issues of generational cultural change and the social identifications, national allegiances and interactions with notions of citizenship and belonging, particularly among the younger generations born within the host countries (*see* Gibson 1988; Ranger, Samad and Stuart 1996). The predominance of issues faced by those immigrants or sections of the ethnic minority communities who are still within the working age or adolescents reaching adulthood, has meant at worst a significant neglect of the particular situations and experiences of the older migrants or those reaching old age in their host societies within the race and ethnicity literature. At best, it has led to attention being focused within the gerontological literature (until at least very recently) mainly on the problematic aspects of ethnic minority ageing.

While the gerontological literature on ethnic minority older people, with its predominant focus on health and care issues as mentioned above, either explicitly or implicitly constructs the ‘ethnicity’ or ethnic ‘culture’ of these older people as a problem, future gerontological research needs to move away from an over-emphasis on ethnicity (especially when it is conceptualised as being synonymous with a bounded culture) as a primary defining feature of old-age experiences for these ethnic minority older people. In particular, there is a need for future research and scholarly work in the area to challenge essentialist notions of ethnicity and to engage more critically with notions of ethnic ‘difference’, recognising instead the instability of ‘ethnicity’ as a fixed and discrete category, much like other social categories including those relating to age, gender and social class. As Elrick, Schneiderhan and Khan note:

We do not argue that race and ethnicity are unimportant or that the results of previous studies are irrelevant. Instead … that these are not necessarily the most important social locations underlying the ‘meaning’ of being a person defined externally (by the state and/or academia) as ethnic … First, rather than impose a priori categories upon research subjects, we should construct them from within real-world interactions and situations. Second, a priori categories often intersect with one another in ways that do not allow for a clean disembedding, so understanding one social category requires that multiple categories be examined simultaneously; this is largely a reiteration of what intersectionality scholars have been arguing for over the last two decades. (2014: 1186–7)

## Conceptualising ethnicity, difference and *otherness* in gerontology – overview of papers

The six papers in this special issue are all written from a non-essentialist theoretical standpoint on ethnicity and ethnic minority ageing – with a particular focus on the fluid, situational, intersectional, relational and changing character of ethnicity and an emphasis on highlighting the heterogeneity of experiences existing within the otherwise predefined, and thus perceptibly fixed, ethnic and age-based categories. The special issue begins with two theory-based, conceptual papers which focus on the current state of the social gerontological literature involving research on ethnic minorities' ageing and later-life experiences. These contributions aim to highlight both the gaps in current knowledge and understandings and the related biases and assumptions within the current research agendas and theoretical frameworks that dominate scholarly thinking within the field of social gerontology with respect to ethnicity and ethnic minority later life and ageing.

In the first of these contributions, Christopher Phillipson (2015) begins the discussion by providing the *Ageing & Society* readership with a critical overview of the documented history of ethnic minority ageing within the social gerontological research literature. He notes how ethnic minority ageing remains a neglected area within social gerontology. This is despite the increasing importance of making ethnicity and ethnic minority ageing core areas of work in social gerontology, especially with the rise of globalisation and transnational communities which, according to Phillipson, have redefined the character of urban neighbourhoods and communities and redefined social relationships and identities within and beyond nation states. Taking the view of ‘ethnicity’ as a dynamic, ongoing process of self-identification and differentiation, Phillipson observes that ethnicity can be seen as a reflexive category linked to global economic and social processes. In this respect, he argues, a focus on minority ethnic ageing within social gerontology can provide a unique approach to thinking about the relationship between age and social structure, and for understanding and documenting the broad range of influences on people (and on their subsequent later-life and older-age experiences) as they move through the lifecourse. Having highlighted the importance of placing ethnicity at the centre of studies of later life and ageing, Phillipson also outlines the ways in which this may be most appropriately achieved within social gerontology, namely by: moving away from largely descriptive research about the health and social problems associated with ethnic group membership; recognising the interaction between ethnicity and other key social statuses of individuals; avoiding a discourse inflected with notions of ‘burden’ and ‘dependency’, recognising instead that older people are engaged with communities in diverse and complex ways; and bringing the awareness back into social gerontology regarding the interlocking nature of age-based and ethnic-based discrimination that ethnic minority older people continue to experience.

Drawing on the theoretical developments in mainstream ethnicity scholarship, Sandra Torres' (2015) contribution that follows further highlights the key gaps and limitations in our current gerontological understandings and conceptualisations with respect to ethnicity and ethnic minority ageing. Presenting a review of the contemporary gerontological research on ethnicity, Torres points out how this body of research appears to be informed predominantly by essentialism and structuralism to the exclusion of the social constructionist perspective and notions of intersectionality which are, otherwise, widely accepted and used within the mainstream ethnicity scholarship. On the part of social gerontology, this lack of recognition of (and engagement with) the more recent theoretical developments, according to her, has far-reaching implications for how ethnic minority older people continue to be perceived and understood in research as well as more generally – often in *othering* terms. Furthermore, this has implications also for how policies of old age are, in turn, formulated and how gerontological practice is shaped in relation to ethnic minorities. Focusing a critical eye on the restrictive and marginalising theoretical frameworks currently in use within gerontological research, Torres emphasises the need for gerontology scholars to expand the gerontological imagination and seriously address how the perspectives adopted by gerontology at present construct ethnic *others*.

The theme of ethnic minority older people's marginalisation and *othering* within gerontological research is further developed and reinforced in Maria Zubair and Christina Victor's (2015) paper. This reflexive paper, which constitutes one of the two methodological contributions within the special issue, interrogates and challenges some of the dominant ‘ethical’ considerations that are embedded within the formalised research ethics frameworks used in research with older, and ethnic minority older, people. Drawing inspiration from the critical perspectives which have already surfaced within the wider research ethics literature beyond the field of social gerontology (*see* Buckle, Dwyer and Jackson 2010; Coomber 2002; Haggerty 2004; Hammersley 2006, 2009; Hammersley and Traianou 2012; McDonach, Rosaline and Williams 2009; Truman 2003), Zubair and Victor discuss the implicit institutional agendas, as well as ideologies with respect to ethnic minority older people and ageing, which underpin formalised research ethics frameworks and guidelines. Using illustrative examples from their own fieldwork experiences with South Asian older people living in the UK, they further describe the type of marginalising and *othering* fieldwork practices these dominant institutional frameworks and ideologies encourage (even dictate) and the type of unequal research relationships these shape for older, and particularly ethnic minority older, people during the research process. The main emphasis of this discussion centres on highlighting how formalised research ethics frameworks may often work in contradictory ways to disempower and disadvantage older ethnic minority people within the research process, particularly so as these official frameworks consistently perceive of and position these older people in essentialist terms by focusing upon, and over-emphasising, their ethnic and older age ‘difference’ and vulnerability.

Focusing similarly on the fieldwork aspects of undertaking research with older people, but specifically in ‘cross-cultural’ and ‘cross-generational’ contexts, Meriel Norris’ (2015) paper once again takes issue with the dominant essentialist understandings of ‘ethnicity’ and ‘age’ which are, otherwise, prevalent within much of the gerontological literature involving non-White and minority ethnicities. Using critical insights from some of the earlier literature in the area of intersectionality and embodiment in particular, Norris explores their relevance in her own cross-cultural fieldwork encounters as a younger White British woman undertaking research with older women and men in Indonesia. With fewer earlier works within the field of ageing studies which have specifically addressed the interaction of the researcher's racial, ethnic and age identities with those of their older research participants (*see* Wray and Bartholomew 2010; Zubair, Martin and Victor 2012*a*, 2012*b*), Norris' contribution usefully adds to this very small body of existing literature, confirming and extending our understandings developed with respect to the intersectional, fluid, situational and embodied character of these social identities and respective social interactions. Her fieldwork experiences, for example, not only reveal the importance of paying attention to the embodied features of a researcher's identities in undertaking research but also point to the fluctuating character of these embodiments. Such situational making and remaking of the researcher's ethnicity, age, gender and other relevant social identities through embodied performances has previously been documented in ageing research involving ethnic minority older people (*see* Zubair, Martin and Victor 2012*a*, 2012*b*), revealing ‘ethnicity’, ‘age’, ‘gender’, and other social identities more generally, to be relational and situational enactments as opposed to being fixed, static and discrete categories. This earlier literature has also revealed that the presumed shared ‘ethnicity’ of the researcher with their ethnic minority older participants does not necessarily translate into either shared cultural understandings or shared subjectivities between the researcher and their ethnic minority older participants in a straightforward way, given the negotiatory aspect of ethnicity as well as other social identities (Zubair, Martin and Victor 2012*a*, 2012*b*). Norris' contribution further confirms this negotiatory and relational aspect of both ethnicity and age identities (as well as other social identities of the researcher and the participant) by revealing, conversely, the meshing of the younger White British female researcher's and her older Indonesian female and male research participants' inter-subjectivities across the apparent ethnic and age-related boundaries.

Having presented in detail some of the key theoretical, methodological and fieldwork-related issues and frameworks linked with ageing research in ethnic minority contexts, the final two contributions that follow present empirical data from studies involving ethnic minority older people living in two very different national and local contexts – namely rural Australia and a small, urban, town in the UK. Both of these studies address the broader topic of health, care provision and health services access among ethnic minority older people. However, the main emphasis in the analysis and discussion remains on the heterogeneity of experiences existing within the broadly defined ethnic categories as well as the key role of both the various social inequalities experienced and differential access to resources, rather than the ethnic ‘culture’ itself, in defining the particular later-life and ageing experiences of these older people from minority ethnic backgrounds.

The first of the empirical contributions by Harriet Radermacher and Susan Feldman (2015) explores issues of health and wellbeing for ethnic minority men of Albanian, Macedonian, Italian and Turkish origins growing older in a rural community in Australia. The paper identifies multiple disadvantages and barriers these men face in relation to ageing well and accessing appropriate health services, for example through being male, living in a rural location, working in physically demanding jobs, occupying their specific social and socio-economic position, and so on. Focusing upon and presenting the own voices and experiences of these ethnic minority men in addition to those of the health service providers, this contribution brings into question the often taken-for-granted (and negative) positioning of ethnic minority older people within the gerontological health research literature in terms of their perceived ethnic *otherness* – *e.g.* as non-literate, uneducated, non-English-speaking *others*, with unhealthy cultural lifestyles, a lack of awareness of health-promoting behaviours and an inability to navigate the health-care system. Radermacher and Feldman argue instead that despite such dominant perceptions among service providers about these men's (ethnic) ‘difference’ being a barrier to their healthy ageing, the men's own experiences reveal not only diversity in their particular circumstances but also a greater range of healthy behaviours and understandings about health promotion than is often assumed. Moreover, contrary to the dominant discourses of health service providers (as well as those prevalent more generally within academia), which continue to frame implicitly these ethnic minority men and their assumed ethnic cultural difference as problematic in terms of their health outcomes, Radermacher and Feldman further illustrate how these men's family and their ethnic identity, together with their work, are in many ways an important resource helping them to age well. A smaller body of the existing gerontological and health-related literature has already documented such potentially positive and protective aspects of ethnicity in relation to old age health and quality of life in later years for ethnic minority individuals (*see* Acharya and Northcott 2007; Dossa 1994; Guglani, Coleman and Sonuga-Barke 2000; Nazroo *et al.* 2003). Despite this, the predominant use of a more problem-focused approach to studying ethnic minority ageing has nevertheless constructed a largely bleak picture of negative health outcomes in later life for ethnic minority older people, resultant from their perceived ethnic ‘cultural’ differences.

The final contribution in this special issue, by Karan Jutlla (2015), further challenges the dominant perceptions in relation to ethnicity, and more specifically with regards to the reasons for ethnic minority older people's lack of equitable access to health services, that continue to prevail within much of the existing social gerontological literature. Focusing on the Sikh community in Wolverhampton in the UK, Jutlla's paper explores the impact of migration experiences and migration identities on these ethnic minority individuals' particular experiences of both the formal statutory services and of caring for a family member with dementia. Through her analysis of her Sikh research participants' personal histories and experiences of caring for their older family member with dementia, Jutlla illustrates how the actual barriers to service use for her research participants are much more external to the Sikh community than is generally recognised, existing in the form of the perceived and actual racism and structural inequalities experienced by these ethnic minority people in their capacity as immigrants, as opposed to simply being linked with issues of ‘community’ and ‘culture’ in a straightforward way. Furthermore, the social and cultural heterogeneity existing within this otherwise ethnically defined community means that there is also a diversity of experiences in relation to how the inequalities are experienced by members of this community, with some members being better equipped than others to negotiate access to the required services.

## Conclusion – ethnic minority ageing research: issues, absences and futures

Ageing research involving ethnic minorities to date has largely been defined by narrow health-related agendas. With strong current tendencies towards using a heavily problem-focused approach and with its specific focus on the health and care issues of older ethnic minority people, this area of research has constructed the ‘ethnicity’ or ethnic ‘culture’ of these older people as problematic. This special issue is an attempt to challenge such constructions using, for example, some of the theoretical insights and understandings developed within the wider literature on ethnicity. Both Phillipson's and Torres' paper contributions have identified important gaps and biases with respect to the understandings of ‘ethnicity’ within the social gerontological research and literature, including the essentialist thinking with respect to ethnicity and the *othering* ideologies and practices that currently dominate the field. Zubair and Victor's contribution has further illustrated the *othering* ideologies and practices with respect to ethnic minority older people within the current research frameworks and agendas and how these recreate social inequalities for ethnic minority older people within the research process itself. Norris, on the other hand, has highlighted the socially constructed and intersectional nature of ethnicity as well as age and gender identities, such that these can be continuously created, recreated and negotiated during fieldwork encounters. In addition to this, Radermacher and Feldman's and Jutlla's empirical contributions have countered the dominant *othering* ideologies and discourses apparent in much of the gerontological research literature on ethnic minority older people, emphasising instead the heterogeneity in ethnic minority older people's situations and experiences as well as the positive and protective aspects of ethnicity, contrary to popular belief.

Identifying the various absences and gaps within current gerontological research in relation to how ‘ethnicity’ is conceptualised, understood and theorised, the various paper contributions within this special issue point to a number of important directions for future ageing research which involves ethnic minority older people. Firstly, there is a need for the recognition of the wider aspects of ageing and old age among ethnic minority groups, beyond the more problem-focused and hence pathologising attention paid so far to ethnic minorities' ageing. Secondly, the positive or protective aspects of ethnicity, ethnic culture and ethnic group belonging, which reveal how ethnicity can be a useful resource for ethnic minority older people, need to be acknowledged. Thirdly, gerontological research and writing needs to take into account the actual cultural diversity and heterogeneity of experiences existing within ethnic groups, such that ethnic distinctions and categorisations can be de-constructed and understood as being less clear-cut and fixed, but rather as superficial, fluid and changing. Finally, the multiple and interconnected dimensions of social inequalities, experienced by those within the particularly more disadvantaged social locations within ethnic minority groups and over the lifecourse, need to be uncovered. This will reveal the role of multiple social inequalities, as opposed to ethnic culture, in the disadvantages experienced by ethnic minority people in their later life and old age.
